# In Vivo Anchoring Bis‐Pyrene Probe for Molecular Imaging of Early Gastric Cancer by Endoscopic Techniques

**DOI:** 10.1002/advs.202203918

**Published:** 2022-11-27

**Authors:** Qiang Luo, Chaoqiang Fan, Wang Ying, Xue Peng, Yiyang Hu, Zhaohui Luan, Shaosong Ye, Chunli Gong, Yu Huang, Yufeng Xiao, Yang Chen, Malcolm Xing, Lei Wang, Shiming Yang

**Affiliations:** ^1^ Department of Gastroenterology Xinqiao Hospital Army Medical University Chongqing City 400037 P. R. China; ^2^ Chongqing Municipality Clinical Research Center for Gastroenterology Office of Science and Technology of Chongqing No. 2 Xingai road Yubei Chongqing 401147 China; ^3^ Department of Mechanical Engineering, Biochemistry and Medical Genetics University of Manitoba Winnipeg Manitoba R3T 2N2 Canada; ^4^ CAS Center for Excellence in Nanoscience CAS Key Laboratory for Biomedical Effects of Nanomaterials and Nanosafety National Center for Nanoscience and Technology (NCNST) No. 11 Beiyitiao, Zhongguancun Beijing 100190 China

**Keywords:** blue laser endoscopy, early gastric cancer, molecular imaging, angiogenesis

## Abstract

With the development of blue laser endoscopy (BLE) technique, it's often used to diagnose early gastric cancer (EGC) by the morphological changes of blood vessels through BLE. However, EGC is still not obvious to identify, resulting in a high rate of missed diagnosis. Molecular imaging can show the changes in early tumors at molecular level, which provides a possibility for diagnosing EGC. Therefore, developing a probe that visually monitors blood vessels of EGC under BLE is particularly necessary. Herein, a bis‐pyrene (BP) based nanoprobe (BP‐FFVLK‐(PEG)‐RGD, M_1_) is designed, which can target angiogenesis and self‐assemble into fibers in situ, resulting in stable and long‐term retention in tumor. Moreover, M_1_ probe can emit yellow‐green fluorescence for imaging under BLE. M_1_ probe is confirmed to steadily remain in tumor for up to 96 hours in mice transplanted subcutaneously. In addition, the M_1_ probe is able to target angiogenesis for molecular imaging of isolated human gastric cancer tissue under BLE. Finally, M_1_ probe i.v. injected into primary gastric cancer model rabbits successfully highlighted the tumor site under BLE, which is confirmed by pathological analysis. It's the first time to develop a probe for diagnosing EGC by visualizing angiogenesis under BLE, showing great clinical significance.

## Graphical abstract

An M_1_ probe is designed for diagnosing early gastric cancer (EGC) through visualizing angiogenesis under blue laser endoscopy (BLE). M_1_ probe can remain in tumor for up to 96 h in mice transplanted subcutaneously. Moreover, the M_1_ probe is confirmed to target angiogenesis for molecular imaging of isolated human gastric cancer tissue and primary gastric cancer model rabbits under BLE.

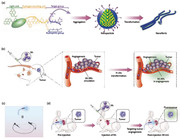

## 1 Introduction

Currently, gastric cancer is one of the most common malignant tumors and has a 5‐year survival rate of only ≈19% at stage IV.^[^ ^1^, ^2^, ^3^ ^]^ Therefore, accurately diagnosing early gastric cancer (EGC) in time can improve patient survival. In recent years, with the development of endoscopic techniques, blue laser endoscopy (BLE) has quickly become one of the most effective methods for the early diagnosis of gastric cancer.^[^ ^4^, ^5^, ^6^ ^]^ Compared with computed tomography (CT), magnetic resonance imaging (MRI) and gastrointestinal radiography, which are traditional methods, BLE with an excitation wavelength range from 410 to 450 nm, is more sensitive to detect EGC by the morphological changes of glandular tubes and blood vessels on the gastric mucosal surface.^[^ ^4^, ^7^, ^8^, ^9^, ^10^ ^]^ Nevertheless, due to the lack of an image probe for vessels or angiogenesis of tumor under endoscopy, there is still an approximate 25% clinical omission diagnostic rate of EGC by BLE, leading to the loss of the best treatment opportunity for gastric cancer patients.^[^ ^7^ ^]^ To satisfy clinical needs, we hypothesized that, a single imaging agent that can visualize the angiogenesis of tumors through BLE shows a great opportunity accurately for aiding clinical doctors to diagnose EGC. A promising agent for tumor angiogenesis of EGC should have the ability (i) the lighting up tumor angiogenesis of EGC under clinical BLE and (ii) stably maintain tumor angiogenesis of EGC in the stomach.

Over the past several years, molecular imaging agents, such as coumarin, rhodamine, and anthocyanidin et al., have been widely used in biological imaging; these agents had the ability to show early changes in cancer at the molecular and cellular levels with high sensitivity and accuracy, which is instrumental in improving the early diagnostic efficiency of EGC.^[^ ^11^, ^12^ ^]^ Despite the extraordinary advances of molecular imaging with respect to the screening of tumors, few techniques were suitable for EGC in the clinic^[^ ^13^ ^]^ which might probably be due to (i) contrast agents' lack of fluorescing ability under BLE; and (ii) the strong acidity of the stomach resulting in short half‐life and instability of agents under the gastric acid environment, which severely restricted its clinical application.^[^ ^14^, ^15^ ^]^ Therefore, approaches to identify through a single molecular probe that could be opportunistically excited for imaging by BLE,^[^ ^16^, ^17^ ^]^ with increased accumulation and prolonged retention time in angiogenesis of EGC by accurately and clinically favorable imaging of cancer lesions.

In our previous work, bis‐pyrene (BP), an aggregation‐induced emission molecule was successfully utilized for tumor imaging and showed great potential in bioimaging.^[^ ^18^, ^19^ ^]^ As a hydrophobic core, BP‐based peptide materials could easily form particles in an aqueous solution by intermolecular *π*‐*π* conjugate stacking.^[^ ^20^ ^]^ Moreover, the most exciting finding is that BP was confirmed to have absorption peaks from 310 to 420 nm, which overlapped with the wavelength of BLE (410–450 nm).^[^ ^4^, ^18^ ^]^ Therefore, the use of BP‐based materials would be a new opportunity to act as an ideal molecular imaging agent of tumor angiogenesis under BLE for efficiently optimizing the diagnostic yield of EGC.

It is well known that agents that targeted aggregation and steadily retained in a tumor is very important for long‐term imaging.^[^ ^21^, ^22^, ^23^, ^24^, ^25^ ^]^ Supramolecular chemistry, a new term in recent years, involved the structural evolution of molecular self‐assemblies via noncovalent interactions supported by the exploration of functional nanomaterials in biomedicine.^[^ ^26^, ^27^, ^28^, ^29^, ^30^, ^31^, ^32^, ^33^, ^34^ ^]^ Being dynamic in nature and presenting responsive behavior associated with supramolecular chemistry,^[^ ^35^, ^36^, ^37^, ^38^, ^39^, ^40^, ^41^, ^42^ ^]^ “in vivo self‐assembly” could be achieved for constructing nanomaterials in situ in vivo with improved biological effects.^[^ ^42^, ^43^, ^44^, ^45^, ^46^ ^]^ Recently, we concentrated on binding‐induced fibrillogenesis of peptide‐based nanomaterials to transform nanofibers with *β*‐sheet structures in situ in vivo for targeting aggregation and efficient retention in tumors.^[^ ^47^, ^48^, ^49^, ^50^ ^]^ Inspired by the advantages of in situ self‐assembled materials,^[^ ^51^, ^52^, ^53^ ^]^ it is supposed that imaging agents can anchor to a tumor marker in the form of stable fibrils in a gastric acid environment for long‐term imaging.^[^ ^48^, ^54^ ^]^ It is well known that peptide sequence RGD can recognize and bind with integrin *α*_v_ *β*_3,_^[^ ^54^, ^55^, ^56^, ^57^, ^58^ ^]^ which is highly expressed in the angiogenesis of many tumors including EGC.^[^ ^59^, ^60^ ^]^ Based on our previous works, RGD was proved to bind with integrin *α*v*β*3 mainly due to the coordination between the carboxyl group in the RGD sequence and the Ca^2+^ in the metal ion chelating adhesion site (MIDAS) of *α*v*β*3 on cell surfaces, subsequently disrupted the original hydrophilic‐hydrophobic balance of peptide‐based nanomaterials and resulted in a morphological transformation.^[^ ^53^, ^54^ ^]^ As a marker of EGC under BLE, angiogenesis with highly expressed *α*v*β*3 provides nutrients for the early growth, metastasis, and invasion of cancer cells in tumors and is an important target_,_^[^ ^61^, ^62^ ^]^ which can act as a target for designing a probe that can anchor on the tumor angiogenesis of EGC and image EGC by BLE.

**Scheme 1:**
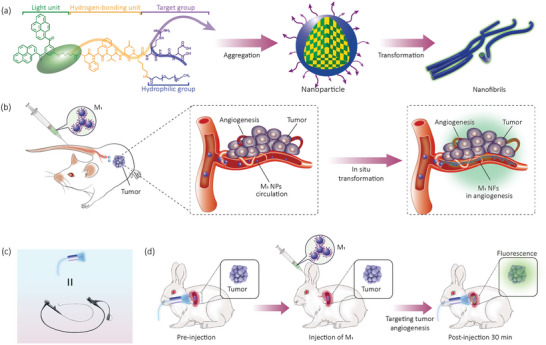
a) The molecular structure and constituent units (fluorescent unit, hydrogen‐bonding unit, target unit, and hydrophilic unit) of M_1_, and fibrillogenesis of M_1_ NPs induced by the binding of ligand‐receptor mechanism; b) Intravenous administration of M_1_ NPs into a model mouse, M_1_ NPs targeted aggregation in the tumor and fibrillated into M_1_ NFs in situ in angiogenesis of tumors for long‐term imaging. c) The schematic picture and image of BLE. d) The M_1_ probe for molecular imaging of model rabbit of primary gastric cancer by BLE.

In order to fulfill clinical needs, herein, we designed a BP‐peptide‐based nanoprobe that could accumulate to angiogenesis of the tumor and detect EGC by molecular imaging under BLE. The BP‐peptide consisted of four motifs: (i) aromatic BP served as a hydrophobic nucleus, (ii) Phe‐Phe‐Val‐Leu‐Lys (FFVLK) sequence originating from amyloid protein (A*β*),^[^ ^63^ ^]^ acted as the frame for the *β*‐sheet nanostructure by forming hydrogen‐bonds, (iii) a short polyethylene glycol (PEG, Mw = 368 Da) chain improved the biocompatibility, and (iv) a target motif RGD sequence also acted as the ligand for binding‐induced fibrillogenesis (**Scheme** **1**a). The nanoprobe (M_1_) based on BP‐FFVLK‐(PEG)‐RGD initially formed stable nanoparticles (NPs) through *π*‐*π* interactions in phosphate‐buffered solution (PBS) with green fluorescence originating from BP. M_1_ NPs could self‐assemble into nanofibers (NFs) induced by chelating Ca^2+^ via a ligand‐receptor binding mechanism (Scheme 1a). The above M_1_ NPs were first i.v. injected into the tumor‐bearing BALB/c nude mice, which could target accumulation in the angiogenesis in tumors and fibrillate to M_1_ NFs, resulting in efficient retention and long‐term imaging up to 96 h (Scheme 1b). BLE we used for experiments was schemed and imaged as Scheme 1c. Additionally, the M_1_ probe was also successfully used to target imaging in human‐isolated tumor tissues by BLE. Finally, the M_1_ probe was applied to primary gastric cancer rabbits, which also realized the identification of gastric cancer by showing an obviously differentiated chromatic aberration between tumor tissue with contiguous normal tissue under the excitation of BLE (Scheme 1d). In the binding‐induced in situ self‐assembly strategy, the binding (targeting) and self‐assembling modules were incorporated and the peptide probe could show targeting ability as well as self‐assembling ability at the same time, promising good imaging performance.^[^ ^48^, ^64^ ^]^ More importantly, we intimately combined the molecular imaging technology and clinical BLE technology in diagnosing EGC, which was able to improve the diagnostic efficiency of EGC because of the imaging ability of the M_1_ probe by BLE. All in one, the M_1_ probe may provide a new opportunity for the molecular imaging diagnosis of EGC under BLE.

## 2 Results and Discussion

**Figure 1:**
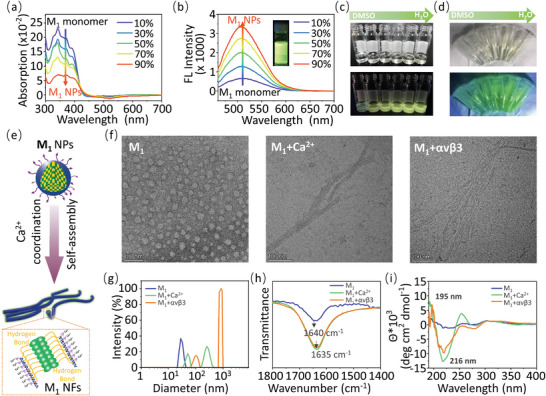
The self‐assembly behavior of M_1_ in solution. a) The UV spectrum analysis and b) the fluorescence assay of M_1_ with different H_2_O contents. An emission peak of M_1_ at 520 nm was detected because of aggregation of the AIE molecule BP. c) The image of M_1_ with different contents of H_2_O under UV lamp irradiation. d) The image of M_1_ with different contents of H_2_O by BLE. e) The schematic illustration of M_1_ fibrillated into NFs triggered by chelation with Ca^2+^. f) The TEM images of M_1_ w/wo Ca^2+^ or *α*v*β*3 at 24 h, respectively. g) The temporal evolution of the size distribution of M_1_ w/wo Ca^2+^ or *α*v*β*3 at 24 h. The FT‐IR spectra h) and the CD spectra i) of M_1_ cultured w/wo Ca^2+^ or *α*v*β*3 at 24 h.

For comparison, molecules M_2_ (BP‐FFVLK‐(PEG)‐RDG) and M_3_ (BP‐GGAAK‐(PEG)‐RGD) were designated as controls (Figure S1 and S2, Supporting Information). The M_1_ quickly precipitated and formed M_1_ NPs when they were dispersed from dimethyl sulfoxide (DMSO) to H_2_O (DMSO/H_2_O = 1/99 in volume), with a decreased UV–vis absorption (**Figure** **1**a) of M_1_ monomers from 310 to 420 nm and increased fluorescence emission at 520 nm (Figure 1b), which was caused by aggregated BP. It was found that the increased fluorescence intensity was observed with increasing water content under ultraviolet (UV) lamp illumination (Figure 1c). Most importantly, M_1_ exhibited a fluorescence trend similar to that of an increase in water via blue lasering imaging (BLI), indicating the potential for molecular imaging of M_1_ under BLE (Figure 1d). The M_2_ and M_3_ controls showed similar optical characteristics to M_1_ (Figure S3, Supporting Information).

In order to validate the fibrillogenesis of M_1_ was mainly triggered by the coordination effect between RGD in M_1_ with MIDAS of *α*v*β*3 (Figure 1e), we first studied the morphology of M_1_ w/wo incubation with Ca^2+^ or *α*v*β*3 for 24 h through transmission electron microscopy (TEM). As shown in Figure 1f and Figure S4 (Supporting Information), M_1_, M_2,_ and M_3_ formed NPs in aqueous solution with diameters of 27.05 ± 4.94, 25.24 ± 2.05, and 25.67 ± 2.57 nm, respectively. Meanwhile, M_1_ fibrillated into NFs with a diameter of 8.33 ± 0.83 or 10.53 ± 0.33 nm after incubation with Ca^2+^ or *α*v*β*3 for 24 h, respectively (Figure 1f). Meanwhile, the control groups M_2_ and M_3_ treated by Ca^2+^ or *α*v*β*3 retained NPs with sizes ranging from 21.49 ± 1.44 to 27.22 ± 1.10 nm (Figure S4, Supporting Information) in 24 h. In order to verify the gradual fibrillogenesis process of M_1_ with time, we also studied the morphology transformations of M_1_ incubated with Ca^2+^ or *α*v*β*3 in solution for 8 and 16 h through TEM. The M_1_ was found to form short nanorods after co‐culturing with Ca^2+^ or *α*v*β*3 for 8 h. Subsequently, they grew into longer fibers at 16 h with a fiber length of 77.38 ± 37.67 and 85.58 ± 29.64 nm, respectively (Figure S5, Supporting Information), which was obviously shorter than those at 24 h, indicating the fibrillogenesis of M_1_ induced by ligand‐receptor binding was a gradual process with time. Additionally, dynamic light scattering (DLS) showed a size range from 30.78 ± 5.01 nm to multi‐disperse upon incubating with Ca^2+^ or *α*v*β*3 (Figure 1g). However, M_2_ and M_3_ treated with Ca^2+^ or *α*v*β*3 revealed no obvious size changes because of the absence of hydrogen‐bond units or target units, respectively (Figure S6, Supporting Information). On the other hand, the TEM images of M_1_ incubated with different metal ions (Ca^2+^, Na^+^, K^+^, Fe^2+^, and Mg^2+^) showed that the Na^+^, K^+^, or Fe^2+^ treated M_1_ held NPs while transformed into NFs under Ca^2+^ or Mg^2+^ (Figure S7a–d, Supporting Information), further indicating that the fibrillogenesis of M_1_ was driven by the coordination effect of RGD with Ca^2+^ ions. Moreover, when co‐incubated with Ca^2+^ and EDTA, a divalent metal chelating agent, M_1_ could not self‐assemble into NFs because EDTA competitively chelated with Ca^2+^, further confirming that the fibrillogenesis of M_1_ was caused by the coordination between Ca^2+^ and RGD (Figure S7e, Supporting Information).

Moreover, we confirmed the secondary structure of M_1_ through Fourier transform infrared spectroscopy (FT‐IR) and circular dichroism (CD). After M_1_ was incubated with Ca^2+^ or *α*v*β*3 for 24 h, the stretching vibration of C = O shifted from 1640 to 1635 cm^−1^ (Figure 1h), indicating that hydrogen bonds were formed in the internal structure of M_1_ NFs. However, no altered C = O stretching vibrations were observed in any of the M_2_ and M_3_ groups (Figure S8, Supporting Information), suggesting that M_2_ and M**_3_** held the morphology of NPs in the presence of Ca^2+^ or *α*v*β*3. CD experiments showed a random coil secondary structure when M_1_ existed alone, but a typical *β*‐sheet structure appeared after M_1_ was cultured with Ca^2+^ or *α*v*β*3 for 24 h (Figure 1i). In contrast, the M**_2_** and M_3_ presented random coil structures (Figure S9, Supporting Information), corresponding to the results of the FT‐IR measurements.

**Figure 2:**
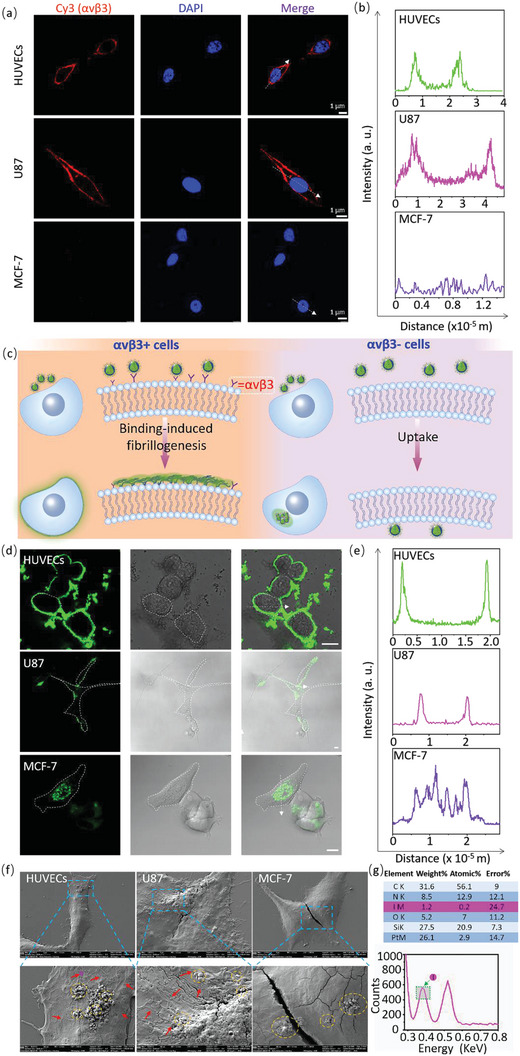
The anchoring of M_1_ on HUVECs. a) CLSM images of immunofluorescence labeling of *α*v*β*3 on the membrane of HUVECs, U87 cells and MCF‐7 cells (E_x_ = 552 nm, collections = 565–610 nm). Scale bar: 1 µm. b) The quantification results of red fluorescence along the white dashed arrow in (a). c) The schematic illustration of M_1_ fibrillated into NFs on *α*v*β*3+ cell membrane and uptaken into *α*v*β*3‐ cells; d) The CLSM images of HUVECs, U87 cells and MCF‐7 cells incubated with M_1_ NPs for 24 h (E_x_ = 405 nm, acquisition range = 450–540 nm). Scale bar: 10 µm. e) The quantification results of green fluorescence along the white dotted arrow in (d). f) The SEM images of HUVECs, U87 cells, and MCF‐7 cells incubated with M_1_ NPs for 24 h. g) The EDS spectrum of the fibers on HUVECs (purple circle in f) and its quantified data, showing the presence of iodine on HUVECs, indicating that the fibers were M_1_ NFs.

To verify the in situ fibrillogenesis and imaging capability of the M_1_ probe at the cellular level due to *α*v*β*3 receptors, we chose human umbilical vein endothelial cells (HUVECs) that overexpressed *α*v*β*3 as the experimental cell line. For comparison, U87 and MCF‐7 cells were selected for use as the positive and negative cell lines, respectively. We first measured the expression of *α*v*β*3 on HUVECs, U87 cells, and MCF‐7 cells through immunofluorescent staining. As shown by confocal laser scanning microscopy (CLSM), an obvious *α*v*β*3 signal (red) was observed on the membrane of HUVECs and U87 cells, while MCF‐7 cells showed no *α*v*β*3 signal (**Figure** **2**a). Additionally, the quantification of the fluorescence signal along with the white dotted line in Figure 2a indicated that *α*v*β*3 expressed on the cell membrane instead of in the cytoplasm and nucleus (Figure 2b). Second, the cytotoxicity of the M_1_ probe on HUVECs was measured through the Cell Counting Kit‐8 (CCK‐8) assays. As shown in Figure S10a (Supporting Information), the M_1_ probe showed no significant cell cytotoxicity to HUVECs at a concentration lower than 40 µM in 24 h, of which the cell viabilities were higher than 86.36% ± 1.35%. Similarly, no obvious cytotoxicity of M_2_ and M_3_ was found with a concentration of up to 40 µM (Figure S10b and S10c, Supporting Information). Moreover, the cell viabilities of HUVECs treated with M_1_‐M_3_ NPs were basically unchanged over time (Figure S10d–f, Supporting Information). The above results indicated the good biological safety of M_1_ NPs to be a probe. Therefore, the concentration of 20 µM was selected for the following studies in vitro.

We schemed the *α*v*β*3 anchoring fibrillogenesis of M_1_ NPs in situ on the membrane (Figure 2c). M_1_ NPs would transform into nanofibers on *α*v*β*3‐positive cells and be easily internalized by *α*v*β*3‐negative cells. To verify the target and fibrillogenesis, HUVECs, U87 cells and MCF‐7 cells were cultured with M_1_ NPs (20 µM) for 24 h, followed by CLSM detection. For *α*v*β*3‐positive HUVECs and U87 cells, green fluorescence was mainly distributed on the cell membrane (Figure 2d). In contrast, M_1_ NPs mainly entered MCF‐7 cells with a large green signal in the cytoplasm after incubation of 24 h (Figure 2d), suggesting that M_1_ NPs could not form NFs on the *α*v*β*3‐negative cell membrane. Moreover, the fluorescence quantification from the membrane‐cytoplasm‐membrane (white dotted line in Figure 2d) further demonstrated that the green signal was merged with the HUVECs/U87 membrane and MCF‐7 cytoplasm, respectively (Figure 2e). These results revealed that M_1_ NPs could adhere to the surface on HUVECs and U87 cells owning to fibrillogenesis, but entered MCF‐7 cells, indicating that *α*v*β*3 drove the fibrillogenesis on the membrane. In addition, when HUVECs, U87 cells, and MCF‐7 cells were treated with M_2_‐ or M_3_ NPs, there was no green fluorescence on the membrane but in the cytoplasm (Figure S11, Supporting Information), further implying the uptake of M_2_ or M_3_ by the cells. The results confirmed that the M_1_ probe was anchored on the cell membrane in the form of nanofibers modulated by RGD‐*α*v*β*3 interaction and KLVFF hydrogen‐bonding units. In order to further verify M_1_ could quickly transform into nanofibers and stably anchored on HUVECs membrane, M_1_, M_2_ and M_3_ incubated with HUVECs for 4 and 8 h were also detected by CLSM. As shown in Figure S12 (Supporting Information), a large number of green fluorescence signals were observed on the cell membrane of M_1_‐treated HUVECs for 4 and 8 h. Meanwhile, weak green fluorescence signals were found inside the cytoplasm of M_2_ and M_3_‐treated HUVECs for 4 and 8 h (Figures S13–S14, Supporting Information). The results indicated that M_1_ could indeed quickly anchor HUVECs membrane and self‐assemble into nanofibers in situ, resulting in long‐term retention on the cell membrane.

We validated the fibrillogenesis induced by *α*v*β*3 on the HUVECs membrane by scanning electron microscopy (SEM). M_1_‐, M_2_‐, and M_3_ NPs (20 µM) were used to culture HUVECs, U87 cells, and MCF‐7 cells for 24 h, followed by careful washing with PBS for 3 times. Then, an SEM was developed to observe the cell morphology. As the SEM images shown, a large number of nanofibrils (red arrows) and some nanoparticles (orange dotted circles) were observed on the membranes of M_1_ NPs treated HUVECs and U87 cells, while only a slight of nanoparticles (orange dotted circles) were seen on the MCF‐7 cells (Figure 2f). In the meantime, M_1_ was labeled with iodine (I) and utilized to highlight the samples in the biological background. After treatment with I‐labeled M_1_, we clearly found that the elemental composition of I on the fibrous structures on HUVECs is 1.2% (purple circle in Figure 2f) through energy‐dispersive X‐ray spectroscopy (EDS) (Figure 2g), suggesting the NFs on HUVECs were originated from the fibrillogenesis of M_1_. For comparison, PBS‐, M_2_‐ and M_3_‐treated HUVECs, U87 cells, and MCF‐7 cells, no NFs were observed on the cell membrane while some residual NPs were stained on cells (Figures S15–S17, Supporting Information), which just attached on the cell membrane without internalizing and washing away.

**Figure 3:**
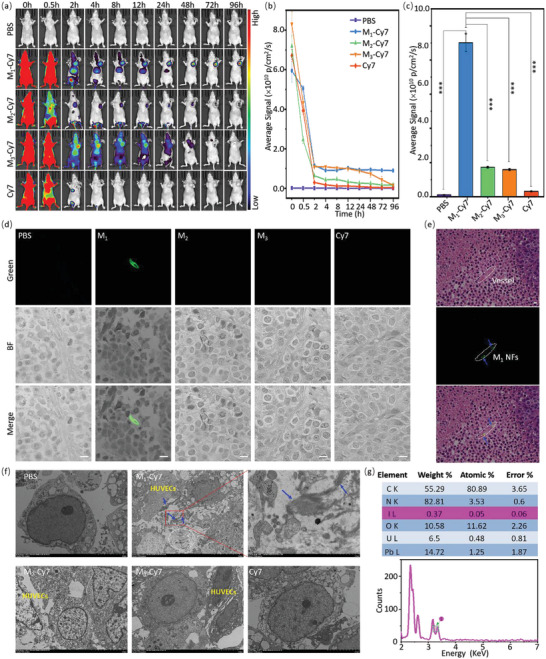
M_1_ enabled long‐term imaging of tumor in xenografted mice. a) Representive in vivo images of Cy7‐labled M_1_‐M_3_ (200 µM, *n* = 3), control Cy7 (20 µm, *n* = 3) and PBS (n = 3) treated BGC‐823‐bearing BALB/c nude mice at 0, 0.5, 2, 4, 8, 12, 24, 48, 72, and 96 h (*λ*_ex_ = 749 nm and *λ*_em_ = 790 nm). b) The quantification of fluorescence intensity from (a). All data were expressed as the mean ± SDs (*n* = 3). c) Average fluorescence intensity of tumor tissue at 96 h. The statistical analysis of data between M_1_ and control groups was measured by two‐sample test (^***^ *P* < 0.001). d) The mice were euthanized at 96 h post‐injection, and the tumors were harvested for sections. The CLSM images of tumor tissue were developed for paraffin sections to detect M_1_ signals. The excitation wavelength was 405 nm, and green channel emission was collected from 450 to 540 nm. Scale bar: 10 µm. e) The tumor of M_1_‐Cy7 mice was stained by H&E and detected by fluorescence microscopy. Scale bar: 10 µm. f) The bio‐TEM images of tumor tissue slices of BGC‐823‐xenografted mice administered glycine labeled iodine (I) M_1_ via i.v. at 96 h. M_1_ NFs (blue arrows) were found in the tumors of M_1_‐Cy7‐treated mice. g) The EDS spectrum of M_1_ NFs in (f) showed 0.37% iodine content, suggesting that the fibers were M_1_ NFs.

BGC‐823 cells xenografted BALB/c nude female mice were chosen to be an animal model to evaluate the long‐term imaging ability of M_1_ in vivo. Before animal experiments, M_1_, M_2,_ and M_3_ were labeled with Cy7‐NHS to obtain M_1_‐, M_2_‐, and M_3_‐Cy7 according to the reaction between the amino group and the NHS group (Figure S18, Supporting Information). M_1_‐Cy7,M_2_‐Cy7, M_3_‐Cy7, Cy7, and PBS with the same dose of 200 µL were i.v. injected into BGC‐823 tumor‐bearing mice. Then, the real‐time biodistribution and tumor accumulation of M_1_‐M_3_ at 0, 0.5, 2, 4, 8, 12, 24, 48, 72, and 96 h post‐injections were measured (**Figure** **3**a). As shown in Figure 3a, the Cy7 fluorescence signals in the bodies of M_1_‐Cy7‐, M_2_‐Cy7‐, and M_3_‐Cy7‐treated mice were much higher than those of the free Cy7 groups at 2 h post‐injection. Furthermore, the Cy7 dyes were almost completely metabolized from the body of free Cy7‐treated mice at 4 h with negligible fluorescence, suggesting that Cy7 conjugation with M_1_‐M_3_ NPs slowed its metabolic rate in the body. We also observed M_1_‐, M_2_‐ and M_3_‐Cy7 targeted aggregation in tumors at 2 h, which was mainly due to the enhanced permeability and retention (EPR) effect of the tumors. The fluorescence signals in the tumors of M_1_‐Cy7 and M_3_‐Cy7 mice were always higher than those of M_2_‐Cy7 mice at the same time period (Figure 3b), mainly because the target effect by RGD within M_1_‐Cy7 and M_3_‐Cy7. In addition, M_3_‐Cy7 mice showed a similar fluorescence change tendency with M_1_‐Cy7 mice before 12 h, but a faster fluorescence decrease in M_3_‐Cy7 groups than M_1_‐Cy7 group after 12 h (Figure 3b). While an apparent red fluorescence was detected in the tumor of M_1_‐Cy7 mice at 96 h, suggesting that M_1_‐Cy7 targeted accumulation in the tumor and in situ transformed into NFs resulting in long‐term retention in the tumor. According to the fluorescence quantification of the tumor site, it was found that the fluorescence in the tumors of the M_1_‐Cy7 group at 96 h was 85.4‐, 5.2‐, 5.7‐ and 28.8‐folds that of the PBS, M_2_‐Cy7, M_3_‐Cy7 and Cy7 groups, respectively (Figure 3c), further confirming the long‐term retention of M_1_‐Cy7. The statistical analysis (t‐test, ^***^ *p* < 0.001) results revealed that M_1_‐Cy7 with binding‐induced fibrillogenesis showed more stable and long‐term imaging signals than controls.

CLSM and bio‐TEM were performed to explore the distribution and fibrillogenesis of M_1_ in tumor tissue. Paraffin sections of ex vivo tumors after post‐injection drug 96 h were obtained and performed by CLSM. It was found that the M_1_‐Cy7‐treated group had an obvious green fluorescent signal in tissue (*λ*_ex_ = 405 nm, *λ*_em_: 450–540 nm), indicating the retention of M_1_ in tumor tissue (Figure 3d). To further verify that M_1_ targeted aggregation in the angiogenesis of tumors, H&E staining was (Figure 3e) measured. The green signal was well located in angiogenesis in tumor tissue (white dotted coil schemed angiogenesis and blue arrows were M_1_ signals, Figure 3e). For bio‐TEM, we observed nanofibrils (blue arrows) nearby HUVECs of angiogenesis in tumor slices of the M_1_‐Cy7‐treated group, but no fibrils in PBS‐, Cy7‐, M_2_‐Cy7‐ and M_3_‐Cy7‐treated mice (Figure 3f). The iodine labeled M_1_ was used to distinguish the M_1_ NFs from the collagenous fibers of tumor tissue (e.g., fibrins in white circle of M_1_‐Cy7 group in Figure 3f) in a biological background. Subsequently, as shown in Figure 3g, the EDS tests on fibrils shown in tumors (blue arrows in Figure 3f) revealed 0.37% elements I composition. Compared with the results from the in vitro experiment, the reduced I content was probably due to the nonspecific absorption of organs or metabolism during drug delivery in vivo. Moreover, the mice were euthanized at 96 h post‐injection, and the major organs were harvested and stained with H&E for pathological analysis. The pathological results (heart, liver, spleen, lung, and kidney tissues) showed no significant differences between M_1_‐Cy7‐treated mice and controls (Figure S19, Supporting Information), indicating that M_1_ was low toxicity and safe without obvious side effects. These results suggested that M_1_ might accumulate in tumor angiogenesis and bind with *α*v*β*3, subsequently self‐assembled into nanofibrils driven by a binding‐induced fibrillogenesis with high accumulation and long‐term retention.

**Figure 4:**
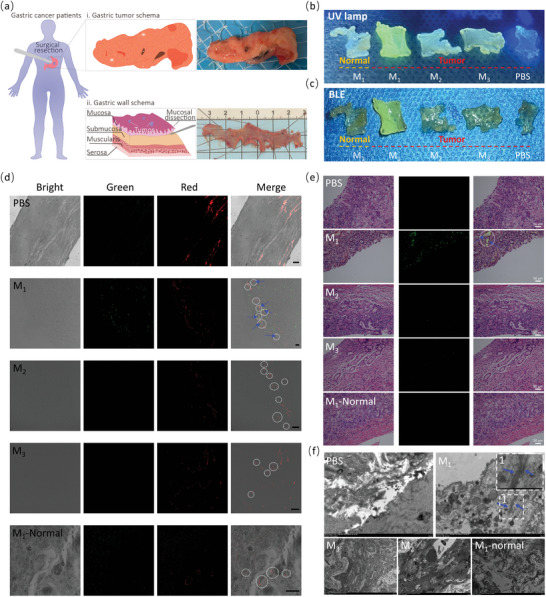
Molecular imaging of clinical resected gastric cancer tissues incubated with M_1_ by BLE. a) Schematic image and physical photo of gastric tumor and gastric mucosa. The gastric tumor was first obtained from surgical resection. The gastric mucosa was then dissected, followed by spreading out with a thumbtack and pretreated with paraformaldehyde overnight. The images of gastric mucosa tissues cultured with M_1_‐M_3_ (50 µM) and PBS under UV lamp (b) and BLE (c) radiation. d) The gastric mucosa tissues from (b)/(c) were used to prepare paraffin sections for analyzing the colocalization of M_1_ NFs and angiogenesis in tumors by immunofluorescence staining. (Green channel, *λ*_ex_ = 405 nm, *λ*_em_ = 450–540 nm; red channel, *λ*_ex_ = 552 nm, *λ*_em_ = 565–610 nm). Scale bar: 25 µm. e) The H&E staining of gastric mucosa tissues from (b)/(c) to observe M_1_ signal in or near angiogenesis, Scale bar: 50 µm. f) The bio‐TEM images of gastric mucosa tissue slices from (b)/(c) to test the fibrillogenesis of M_1_ NPs on the angiogenesis of tumors.

To evaluate the molecular imaging ability of M_1_ for gastric cancer under BLE, gastric cancer tissues and para‐carcinoma tissues were obtained from clinical surgery resection (**Figure** **4**a–i). Then, the gastric mucosal was dissected from the ex vivo gastric tumor followed by spreading out with thumbtacks and immobilized with 2.5% glutaraldehyde solution overnight (Figure 4a‐ii). Thereafter, the tissues were cultured with PBS, M_1_, M_2,_ and M_3_ on a table concentrator at 37 °C for another 24 h, washed with PBS for 3 times, and excited with a UV lamp (*λ*_ex_ = 365 nm) and BLE (*λ*_ex_ = 410–450 nm). Additionally, para‐carcinoma tissue (normal tissue) treated with M_1_ was chosen as another contrast. First, the images of tissues under a UV lamp showed strong green fluorescence on M_1_‐treated tumor tissue, while other controls showed little fluorescence (Figure 4b), indicating that M_1_ can identify and image tumors which is because its target and fibrillogenesis in situ make it to resist the scouring of PBS and improve enrichment and retention. To verify the clinical feasibility of M_1_, tissues excited by clinical BLE were examined (Figure 4c). Compared with the control groups, M_1_ treated mucosa of the gastric tumor showed relatively obvious green fluorescence which corresponded to the UV results (Figure 4b,c). However, the green fluorescence under BLE was much weaker than that under the UV lamp, which mainly occurred because (i) the UV lamp had much higher power than the BLE did, and (ii) M_1_ easily absorbed 365 nm light with the UV lamp for imaging. For all that, M_1_ still has the ability to image gastric cancer through BLE.

To study the target angiogenesis and fibrillogenesis of M_1_ in gastric cancer tissue from surgery, immunofluorescence staining of PBS‐, M_1_‐, M_2_‐ and M_3_‐treated tissues was performed with anti‐CD105 (an maker for tumor angiogenesis) antibody, followed by observation through CLSM. The CLSM images revealed that angiogenesis (red) in tumor tissues was more considerable than that of normal tissue, indicating that angiogenesis was richer in tumor tissues (Figure 4d). We also found that only the M_1_‐treated tumor group showed some green fluorescence signal (blue arrows in Figure 4d), some of which were next to angiogenesis (white dotted circles in Figure 4d), indicating that M_1_ could target angiogenesis, which might be suitable for clinical target detection of gastric cancer. To visualize M_1_ accumulation and fibrillogenesis around angiogenesis easily, H&E staining and bio‐TEM were developed (Figure 4e,f). The H&E staining images showed that the green fluorescence of M_1_ (blue arrows in line 2) was mainly merged with angiogenesis (white dotted circles in line 2) in M_1_‐treated tumor tissue, while M_2_ and M_3_ tumor tissues (line 3 and line 4) and M_1_‐treated normal tissue (line 5) were detected rarely even no green signal (Figure 4e). In addition, the bio‐TEM images clearly revealed that lots of clearly visible nanofibrils were in the M_1_ cultured tumor tissue (blue arrows in Figure 4f and illustration of Figure 4f, but without any fiber in control groups, which was similar to the results of isolated tumor tissue of mice, further indicating the fibrillogenesis of M_1_ was induced by *α*v*β*3 on angiogenesis.

**Figure 5:**
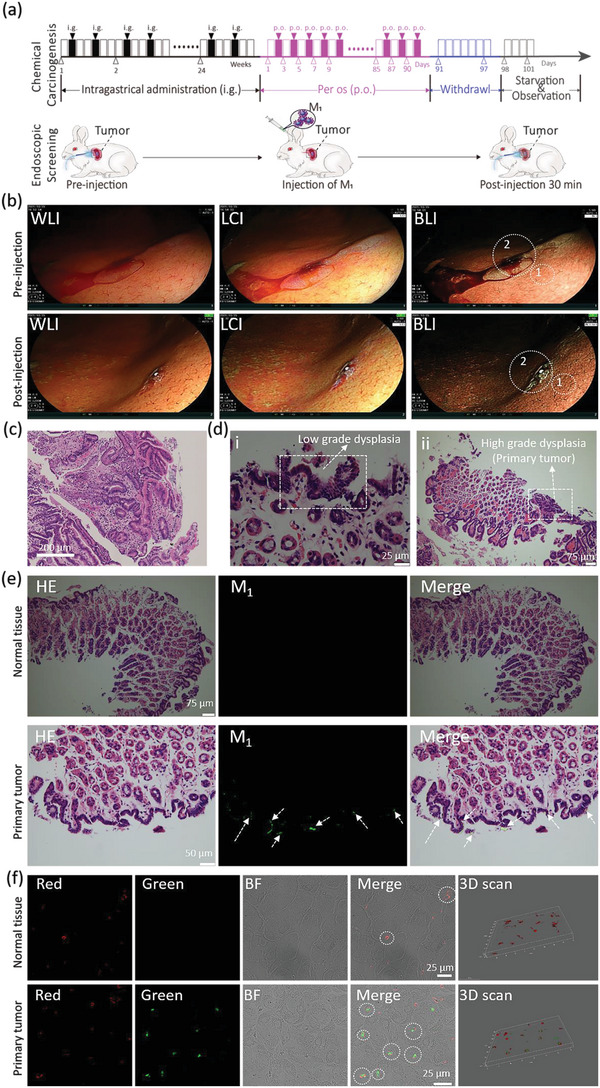
M_1_ enabled molecular imaging of primary gastric cancer in model rabbits by BLE. a) The schematic overview of the model rabbits of primary gastric cancer by chemical agents administered intragastrically (i.g.) with N‐methyl‐N‐nitrosourea (MNU, 10 mg kg^−1^) twice a week for 24 weeks, followed by oral administration of 40 mg of MNU by drinking water every 2 days for another 90 days. Additionally, the schematic of i.v. administration of M_1_ NPs into model rabbits for endoscopic screening under BLE. b) The endoscopic images of gastric tissue under three endoscopic modes (WLI, LCI, and BLI) before and after administration. The biopsy tissues were obtained from gastric tissue for ex vivo H&E staining and pathologic analysis. c) The H&E staining of normal gastric tissue from zone 1 in (b); d) The H&E staining of the lesion site from zone 2 in (b). e,f) The gastric mucosa tissue from zones 1 and 2 in (b) was used to prepare paraffin sections for H&E staining and fluorescence microscope detection (e); and f) immunofluorescence staining for analyzing the colocalization of M_1_ and angiogenesis in tumors by CLSM. (Green channel, *λ*_ex_ = 405 nm, *λ*_em_ = 450–540 nm; red channel, *λ*_ex_ = 552 nm, *λ*_em_ = 565–610 nm).

To evaluate the clinical value and molecular imaging potential of M_1_ in EGC, primary gastric cancer model rabbits were induced with chemical drugs in accordance with an optimization method.^[^ ^65^, ^66^ ^]^ As shown in **Figure** **5**a, the chemical drug N‐methyl‐N‐nitrosourea (MNU) (with a single dose of 10 mg kg^−1^) was i.g. (intragastrical administration) administered twice a week for 24 weeks, followed by oral administration of 40 mg MNU to rabbits by drinking water every 2 days for another 90 days. At the beginning, four rabbits were used to construct the model of primary gastric cancer, but one of them died halfway through administration. For gastroscopy screening, the model rabbits were first fasted for 3 days. For comparison, rabbits were examined at 30 min pre‐ and post‐administration by gastroscopy through the BLE. Before administration, a bulge (white dotted circle 1 in Figure 5b upper) was observed on the surface of the stomach of one rabbit under three endoscopic modes (white light imaging (WLI), linked color imaging (LCI) and BLI of BLE, which was suspected of cancerization (Movie S1 and S2, Supporting Information). Unfortunately, the other two rabbits failed to construct gastric cancer models which were quite normal under BLE screening (Movie S3 and S4, Supporting Information) and were abandoned for subsequent experiments. Then, the suspected cancerous rabbit was administered 2 mL M_1_ NPs (2 mM) via intravenous injection. Subsequently, 30 min after the injection of M_1_, a significant bright yellow‐green fluorescence appeared at the suspected lesion site under BLI mode (white dotted circle 2 in Figure 5b below and Movie S2, Supporting Information). By comparison, no fluorescence was observed on the normal tissue (white dotted circle 1 in Figure 5b below) next to the lesion site (Movie S2, Supporting Information). Considering the target of M_1_ in the angiogenesis of tumors, we hypothesized that the lesion was cancer. To confirm the lesion with fluorescence was cancerous, the tissues in circle 1 and circle 2 after BLI detection were harvested for the preparation of slices, followed by H&E staining and pathological analysis. As shown in Figure 5c, the tissue that came from region 1 was very normal without any pathological changes. However, the tissue of region 2 had some low‐grade epithelial gastric dysplasia, which might have the tendency to develop into cancer with continuous chemical induction (Figure 5d‐i). Moreover, an obvious high‐grade epithelial gastric dysplasia accompanied tissue from region 2 in Figure 5b (Figure 5d‐ii), which was often considered to be the maker of gastric cancer.^[^ ^67^ ^]^ The BLE detection and H&E results indicated that we successfully constructed a rabbit model of primary gastric cancer, and the M_1_ probe was able to guide the molecular imaging of gastric cancer under BLE, showing great potential in EGC detection.

To investigate the fluorescence signal under BLE originating from M_1_, a fluorescence micrography of the tumor overlying the gastric mucosa was developed. As shown in Figure 5e, intense fluorescence (white arrows in line 2) was detected in primary tumor tissue but a negligible signal was detected in normal tissue (line 1). To further confirm the M_1_ targeted tumor angiogenesis, paraffin sections of ex vivo tissue from the primary tumor and normal tissue at 30 min post‐injection of M_1_ were immunofluorescently stained with anti‐CD105 antibody (red channel, *λ*_ex_ = 552 nm, *λ*_em_ = 565–610 nm, Figure 5f). As observed via CLSM, more red signals were detected in the tumor tissue than that in normal tissue, indicating more angiogenesis in tumors. In addition, many green fluorescent signals were observed in primary tumor tissue and nearly all overlapped with angiogenesis (red), demonstrating M_1_ (green) targeted accumulation in angiogenesis due to the high expression of *α*v*β*3 on tumor angiogenesis. The results confirmed that M_1_ had the ability to target tumor angiogenesis for molecular imaging through BLE.

## 3 Conclusion

A BP‐peptide based nanoprobe was verified as a promising molecular imaging probe for EGC under BLE. The probe could target and accumulate in tumors by recognition of *α*v*β*3 on angiogenesis of EGC, followed by transforming into NFs in situ driven by ligand‐receptor interactions with long‐term retention. Most importantly, the probe could emit yellow‐green fluorescence under BLE, representing the first time a molecular imaging probe for BLE was proposed. The in situ self‐assembled probes for BLE showed great potential for the detection of EGC and guiding surgical resection.

## Conflict of Interest

The authors declare no conflict of interest.

## Supporting information

Supporting Information

Supplemental Movie 1

Supplemental Movie 1

Supplemental Movie 3

Supplemental Movie 4

## Data Availability

Research data are not shared.
