# Supplementary material for: In Vivo Anchoring Bis‐Pyrene Probe for Molecular Imaging of Early Gastric Cancer by Endoscopic Techniques
Source: Adv Sci (Weinh). 2022 Nov 27;10(4):2203918. doi: 10.1002/advs.202203918 (PMC9896057; doi:10.1002/advs.202203918)
Supplement: Supplementary file 1 — Supporting Information [file ADVS-10-2203918-s003.pdf]

ADVANCED SCIENCE
Open Access

Supporting Information

for *Adv. Sci.*, DOI 10.1002/advs.202203918

In Vivo Anchoring Bis-Pyrene Probe for Molecular Imaging of Early Gastric Cancer by Endoscopic Techniques

Qiang Luo, Chaoqiang Fan, Wang Ying, Xue Peng, Yiyang Hu, Zhaohui Luan, Shaosong Ye, Chunli Gong, Yu Huang, Yufeng Xiao, Yang Chen, Malcolm Xing, Lei Wang* and Shiming Yang**

Supporting Information

In Vivo Anchoring Bis-Pyrene Probe for Molecular Imaging of Early Gastric Cancer by Endoscopic Techniques

Qiang Luo [a, 1], Chaoqiang Fan [a, 1], Wang Ying [a, 1], Xue Peng [a], Yiyang Hu [a], Zhaohui Luan [a], Shaosong Ye [a], Chunli Gong [a], Yu Huang [a], Yufeng Xiao [a], Yang Chen [a], Malcolm Xing [c, *], Lei Wang [b, *] and Shiming Yang [a, *]

[a] Department of Gastroenterology, Xinqiao Hospital, Army Medical University, Chongqing City 400037, P.R. China

[b] CAS Center for Excellence in Nanoscience CAS Key Laboratory for Biomedical Effects of Nanomaterials and Nanosafety National Center for Nanoscience and Technology (NCNST) No. 11 Beiyitiao, Zhongguancun, Beijing 100190, China.

[c] Department of Mechanical Engineering, Biochemistry and Medical Genetics, University of Manitoba, Winnipeg MB, R3T 2N2, Manitoba, Canada.

[1] Same contribution
[*] Corresponding authors:
Malcolm. xing@umanitoba.ca (MX), wanglei@nanoctr.cn (LW), yangshiming@tmmu.edu.cn (SY).

Materials and Methods

Materials.

The BP-COOH was obtained from Wang Lei's group of the National Center for Nanoscience and Technology. Asp-resin (loading: 0.351 mM/g), Gly-resin (loading: 0.309 mM/g) and Fmoc-amino acids for peptide synthesis were purchased from GL Biochem Ltd. (Shanghai, China), Methoxy poly (ethylene glycol) (mPEG$_6$-CH$_2$CH$_2$COOH) (average M_n = 368.42) was purchased from Biomatrik Inc. (Jiaxing, Zhejiang). MNU) were obtained from Sigma-Aldrich. The Cy7-NHS were obtained from Goyoo Biotech Co. Ltd. αvβ3 protein was purchased from Acro-biosystems (Beijing, China). The anti-CD105 antibody was purchased from Abcam, Cambridge. HUVECs, U87, MCF-7 and BGC-823 cell line obtained from American Type Culture Collection (ATCC). The other solvents and reagents were used as received.

Methods.

The preparation of M_1-, M_2- and M_3 NPs. We first synthesized the molecules **M_1-M_3** through standard solid phase peptide synthesis techniques via Fmoc-coupling chemistry. Then, we studied the structures of **M_1-M_3** through matrix-assisted laser desorption ionization time-off light mass spectrometry (MALDI-TOF-MS, MALDI-2090, Shimadzu Corporation, England). For preparing **NPs**, **M_1-M_3** solid power were firstly dispersed in DMSO with a concentration of 2.0 mM, followed by diluting 100 times with PBS to acquired 20 μM **M_1-, M_2-** and **M_3 NPs**.

UV-vis spectrum analysis and fluorescence assay. M_1-M_3 were dissolved in DMSO (2 mM) and then gradually diluted with water in proportion (V_{water}% = 10%, 30%, 50%, 70% and 90%). And, each stage was detected independently.

TEM measurements for verifying M_1 fibrillogenesis. The **M_1** monomers were decentralized in DMSO (2 mM), and diluted with water to obtain 20 μM **M_1 NPs**. The **M_1** samples with or without Ca^{2+} (20 μM) or αvβ3 (20 μg·mL^{-1}) were detected by TEM (TALOS F200S, Thermo Fisher Scientific, Holland) at hours. The uranyl acetate was chosen as the dye for TEM sample dyeing.

DLS measurements and FT-IR measurements. The prepared **M_1 NPs** were co-incubated with Ca^{2+} (20 μM) or αvβ3 (20 μg·mL^{-1}), followed by detection with dynamic light scattering (DLS, Omni, America) and FT-IR (Spectrum100, PerkinElmer) at 24 h. For comparison, free **M_1 NPs** were also measured.

CD spectra. HFIP was first used to prepare **M_1 NPs** (40μM). And the CD analysis of **M_1 NPs** treated with or without Ca^{2+} (20 μM) or αvβ3 (20 μg·mL^{-1}) were developed by a Circular Dichroism Spectrometer (MOS-450, (Bio-Logic, France)) at 24 h.

Cell Counting Kit-8 (CCK-8**)** assay**.** The CCK-8 assay was performed to explore the cytotoxicity of **M_1-, M_2-** and **M_3 NPs** on HUVECs. Generally, HUVCEs cells were firstly seeded in 96-well plates with a density of 5 × 10^3 cells per well, followed by incubating with complete DMEM medium at a humidified environment containing 5% CO_2 at 37 °C overnight. Then, the medium was moved away and washed with PBS for 3 times, follwed by adding **M_1-M_3 NPs**, which were duilted to 10, 20, 30, 40, 50 and 100 μM with serum free medium, respectively. **M_1-M_3 NPs** were co-cultured with HUVECs for additional 24 h. Subsequently, **M_1-M_3 NPs** were replaced with CCK-8 solution (V_{CCK-8}/V_{DMEM} = 10%) and co-incubated for another 1 h, follwed by CCK-8 assay through a machine of Multiskan GO 1510 (Thermo Fisher Scientific).

Confocal laser scanning microscopy (CLSM) observation. Firstly, HUVECs, U87 cells and MCF-7 cells were seeded in complete media in a humidified atmosphere with 5%

CO_2 and then cultured at 37 °C overnight. Subsequently, all the cells were cultured by 1 mL of fresh serum-free medium containing 20 μM **M_1- M_2-** or **M_3 NPs** for another 24 h and washed with PBS for 3 times. Then, the medium was replaced with PBS and cells were imaged through a Leica Dmi8 CLSM.

Scanning electron microscopy (SEM). HUVECs, U87 and MCF-7 cells were incubated on silicon slice by PBS, **M_1- M_2-** or **M_3 NPs** (20 μM) for 24 h, respectively. Then, above silicon slices with cells were washed with PBS for 3 times, followed by fixing with 2.5% glutaraldehyde overnight. Subsequently, the fixed cells were dehydrated by 50%, 70% and 90% ethanol once for 10 mins, and 100% ethanol twice for 10 mins. Finally, the silicon slice with cells were sprayed with gold for 30 s for SEM measurement by a FIB-SEM microscope (Crossbeam 340, Zeiss).

In vivo evaluation of M_1 self-assembly formation in tumor. The BGC-823 cells (1×10^7/well) were inoculated subcutaneously into the flank of each female BALB/c nude mice (weight 17 ± 2 g). After 6 days, Cy7 labeled **M_1-M_3** (200 μL, 200 μM), free Cy7 (200 μL ,20 μM) and PBS (200 μL) were injected via the tail vein. The images were achieved at 0, 0.5, 2, 4, 8, 12, 24, 48, 72, 96 h post administration by IVIS Lumina III (Perkin Elmer, America).

The evaluation of M_1 NFs for molecular imaging of isolated human gastric cancer tissues under BLE. Firstly, the tissues were obtained by surgical resection of the patients with gastric cancer after the patient's agreements. Then, the mucosal layer of gastric cancer tissue was stripped out and fixed with paraformaldehyde for 12 hours. Then, the paraformaldehyde was replaced by 1 mL PBS solution containing **M_1- M_2- and M_3 NPs** (50 μM) and incubated on a shaking table at 37 °C for another 24 hours. Afterwards, tissues were washed with PBS for three times, followed by imaging through BLE.

In vivo evaluation of M_1 nanoprobe for molecular imaging under BLE on primary gastric cancer model rabbit. Primary gastric cancer rabbits were induced by N-methy-N-nitrosourea (MNU), a chemical carcinogens for gastric cancer. Firstly, a single dose of 10 mg/kg were administered into 6 weeks old rabbits via gavage twice a week for 24 weeks, followed by oral administration of 40 mg MNU to rabbits via drinking water every 2 days for another 12 weeks. For BLE observation, the model rabbits were fasted for 3 days, followed by detecting and imaging pre- and post-injection 30 mins **M_1 NPs**.

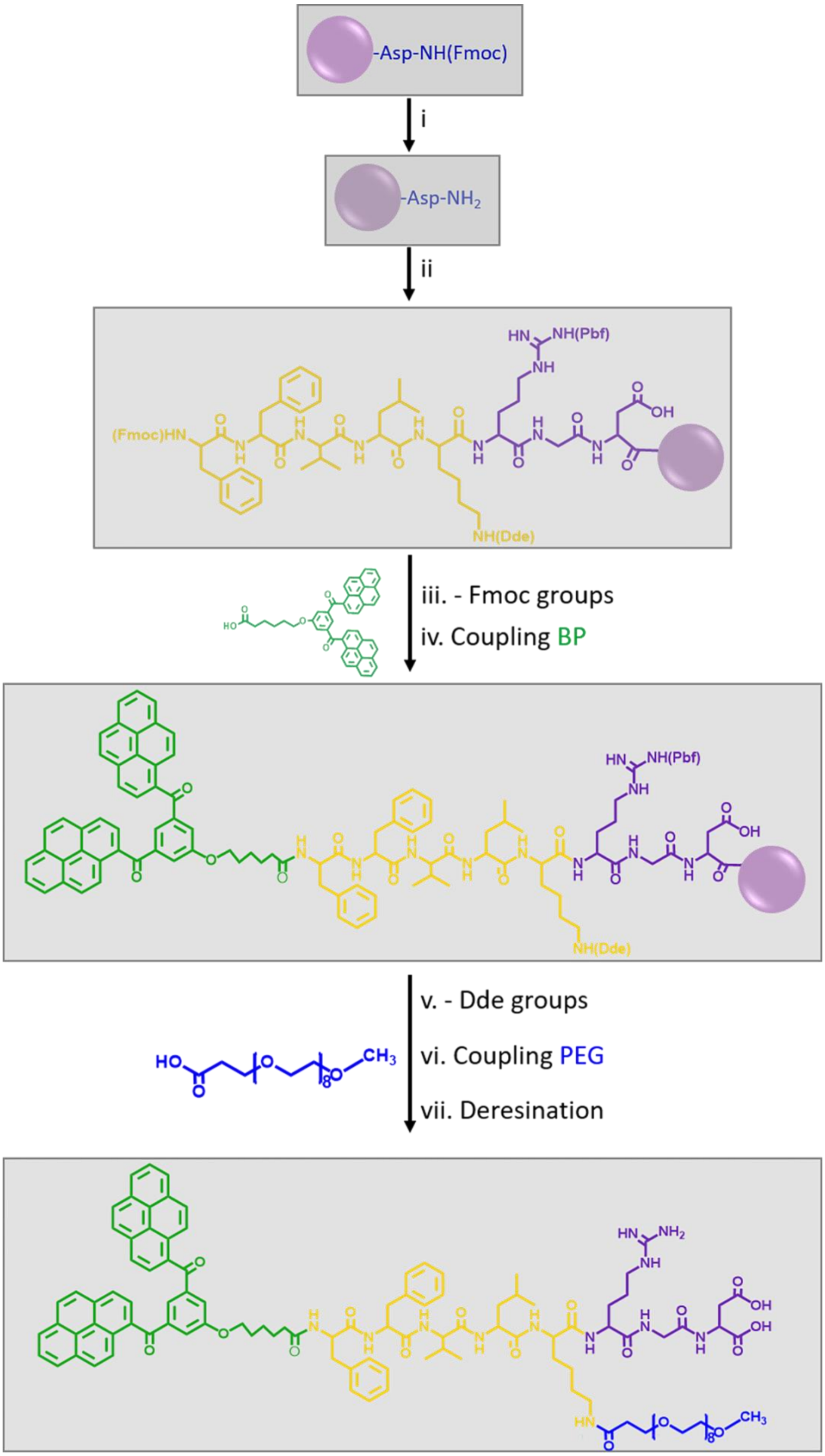

Figure S1. The synthetic route of $\mathbf{M_1}$ by solid phase synthesis method. (i) 25% piperidine in DMF for 10 min; (ii) Fmoc solid-phase peptide synthesis as the designed sequence: Fmoc protected amino acids, HOBT and HBTU, 2 h; (iii) 2% DBU in DMF (iv) bi-pyrene (BP), NMM, HBTU, HOBT, DMF, 12 h (v) 2% $N_2H_4{\cdot}H_2O$ in DMF; (vi) polyethylene glycol (PEG_{368}), HOBT and HBTU, 12 h; (vii) TFA/TIS/H2O (v/v/v = 95:2.5:2.5), 2.5 h.

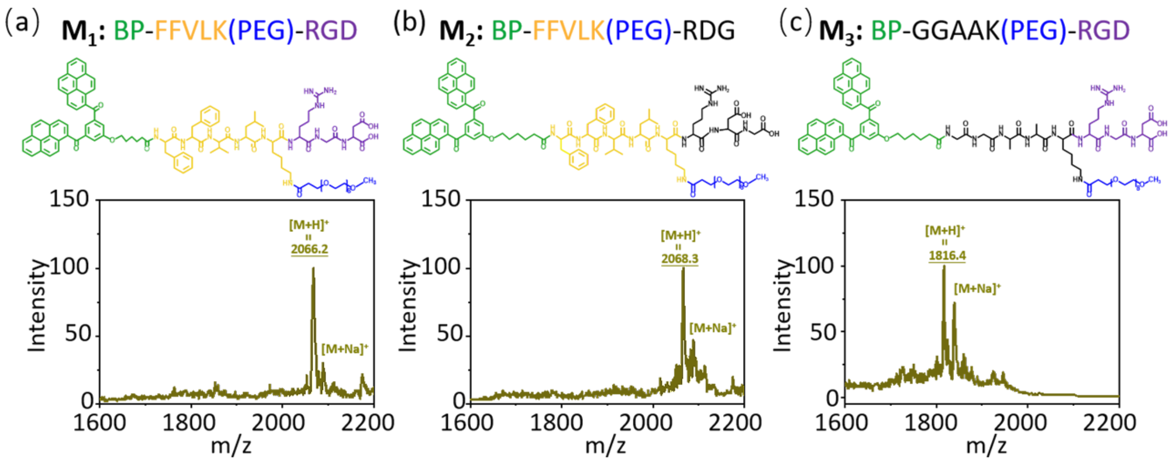

Figure S2. The structure diagram and the MALDI-TOF mass spectrum of $\mathbf{M_1}$ (a), $\mathbf{M_2}$ (b) and $\mathbf{M_3}$ (c).

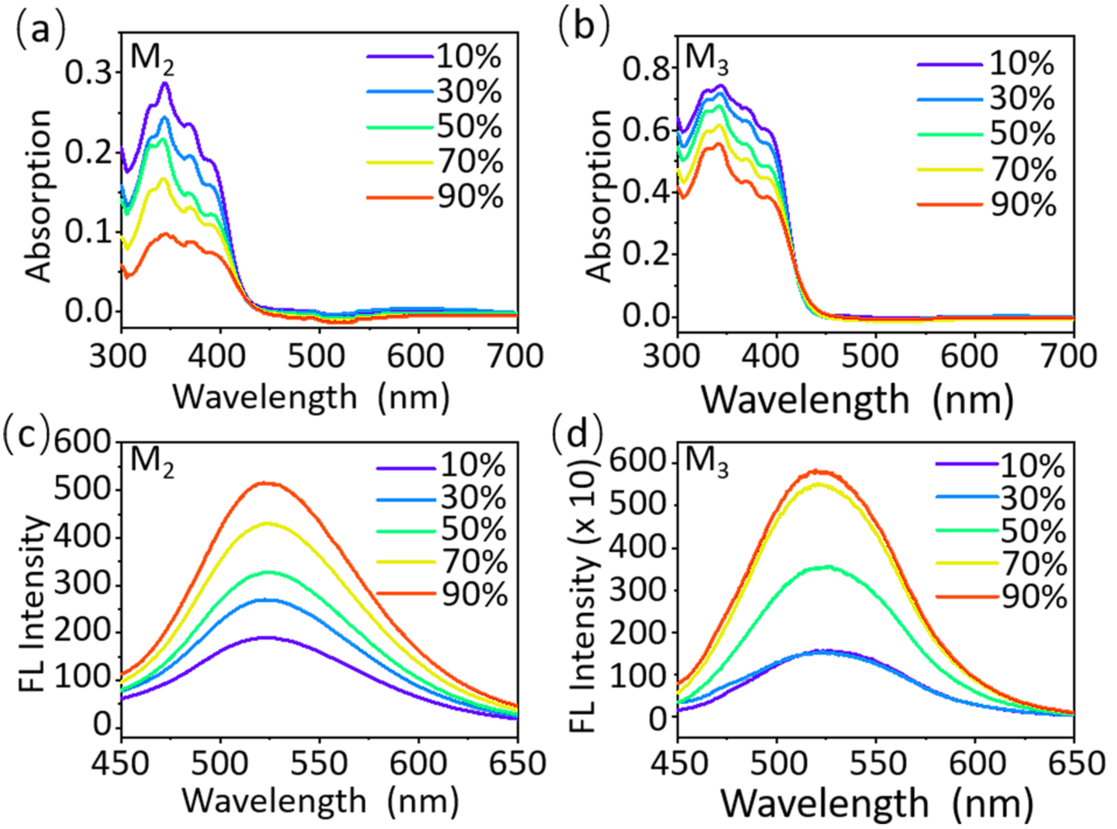

Figure S3. The UV-vis spectrum analysis of $\mathbf{M_2}$ (a) **and** $\mathbf{M_3}$ (b), the absorption peak of $\mathbf{M_2}$ or $\mathbf{M_3}$ at 410 nm decreased gradually with the increase of H_2O content, which was caused by the aggregation of monomers into nanoparticles. The fluorescence assay of $\mathbf{M_2}$ (c) **and** $\mathbf{M_3}$ (d), with an emission peak of $\mathbf{M_2}$ or $\mathbf{M_3}$ at 520 nm because of AIE molecule BP.

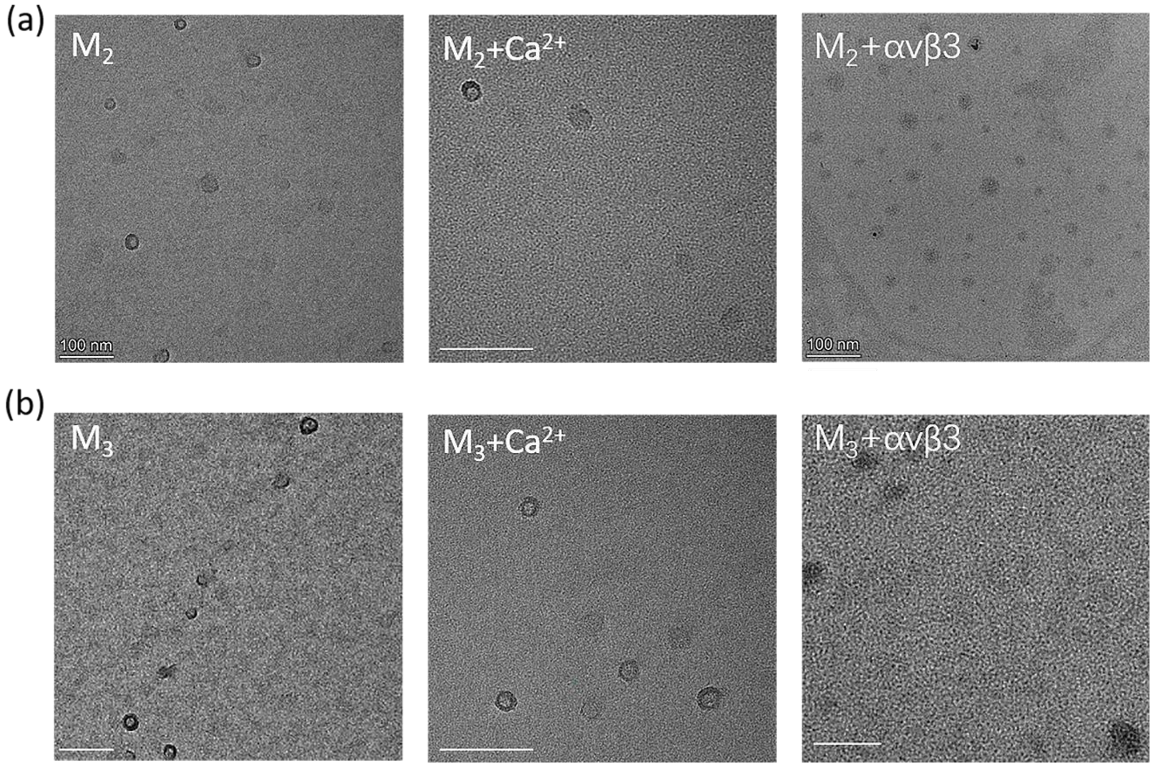

Figure S4. (a) The TEM images of $\mathbf{M_2}$ with or without Ca^{2+} or αvβ3 at 24h; (b) The TEM images of $\mathbf{M_3}$ with or without Ca^{2+} or αvβ3 at 24h. Scale bar: 100 nm.

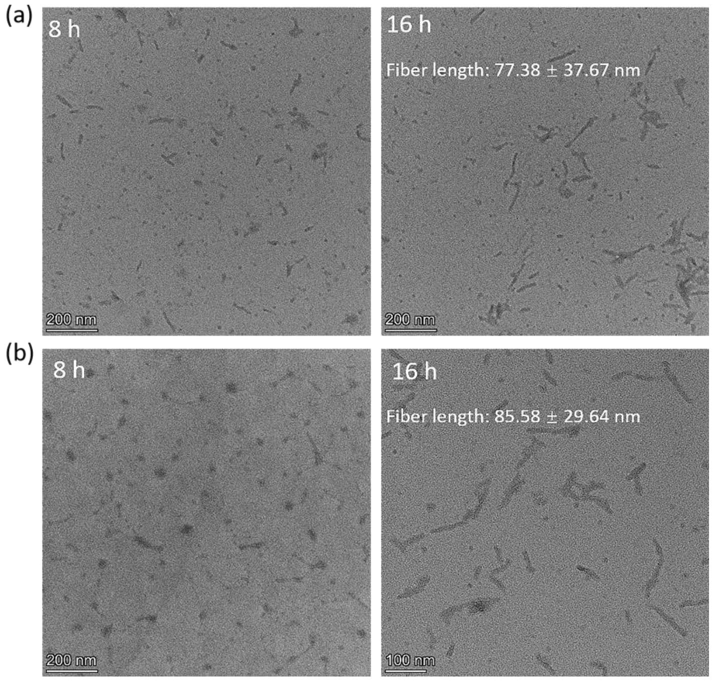

Figure S5. (a) The TEM images of $\mathbf{M_1}$ treated with Ca^{2+} (a) or αvβ3 (b) for 8 h and 16 h. The fiber length of $\mathbf{M_1}$ treated by Ca^{2+} or αvβ3 for 16 h were 77.38 ± 37.67 nm and 85.58 ± 29.64 nm, respectively.

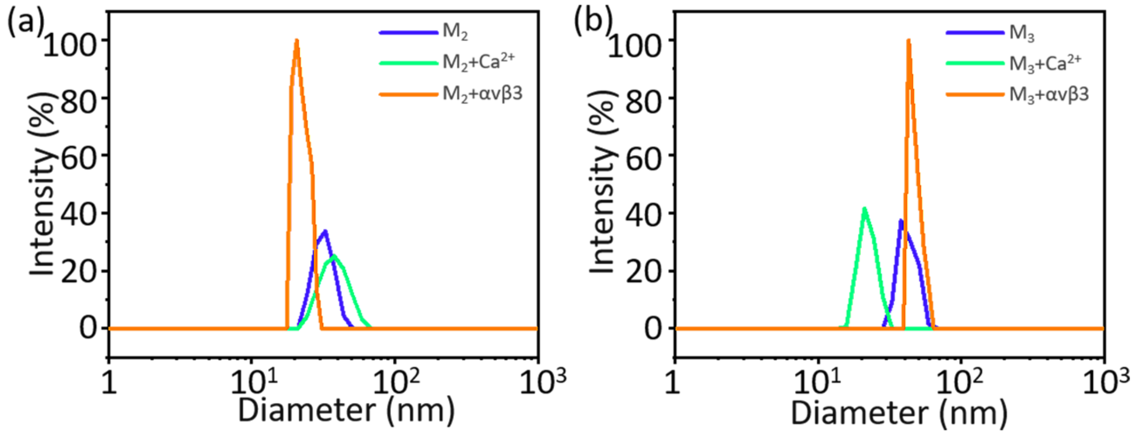

Figure S6. The temporal evolution of the size distribution of $\mathbf{M_2}$ (a) and $\mathbf{M_3}$ (b) with or without Ca^{2+} or αvβ3 at 24h, respectively;

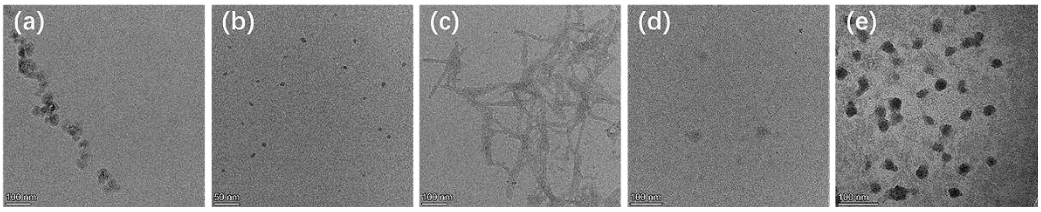

Figure S7. The TEM images of $\mathbf{M_1}$ treated with different metal ions, Na^{+} (a), K^{+} (b), Mg^{2+} (c) and Fe^{2+} (d), the concentration of metal ions was 20 μM. (e) $\mathbf{M_1}$ was co-incubated with Ca^{2+} (20 μM) and EDTA (20 μM) for 24 h, and $\mathbf{M_1}$ held nanoparticles.

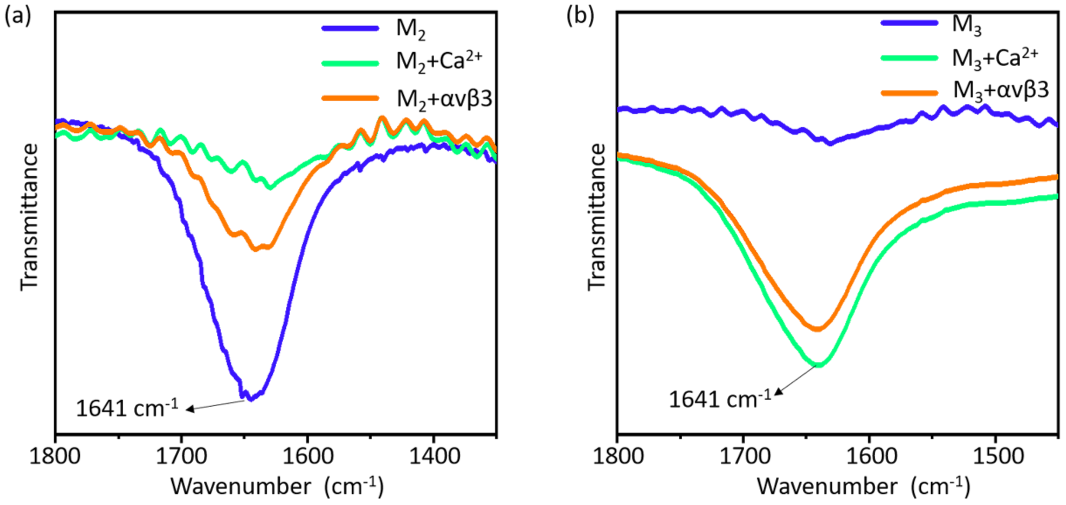

Figure S8. Characteristic absorption bands of infrared spectroscopy measurements of $\mathbf{M_2}$, $\mathbf{M_2}$+Ca^{2+} and $\mathbf{M_2}$+αvβ3 (a), Characteristic absorption bands of infrared spectroscopy measurements of $\mathbf{M_3}$, $\mathbf{M_3}$+Ca^{2+} and $\mathbf{M_3}$+αvβ3 (b).

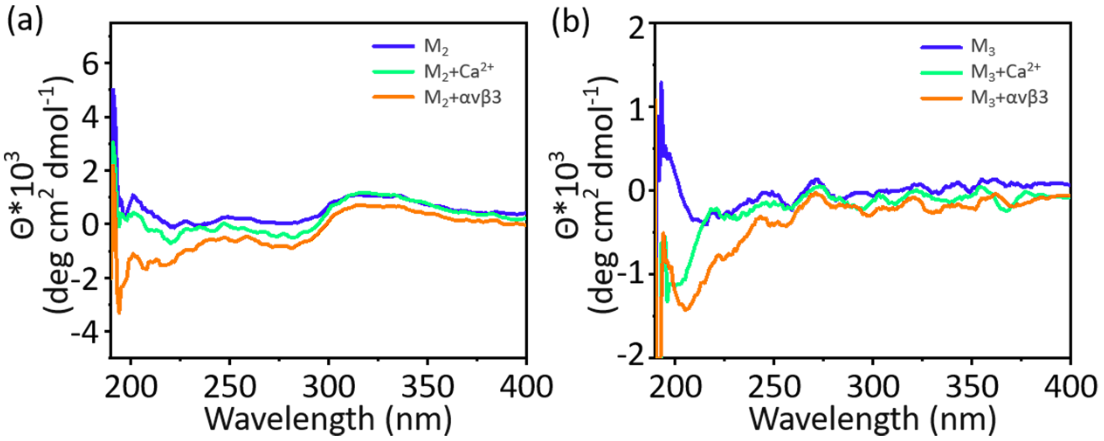

Figure S9. The circular dichroism (CD) measurements of $\mathbf{M_2}$, $\mathbf{M_2}$+Ca^{2+} and $\mathbf{M_2}$+αvβ3 (a), The circular dichroism (CD) measurements of $\mathbf{M_3}$, $\mathbf{M_3}$+Ca^{2+} and $\mathbf{M_3}$+αvβ3 (b).

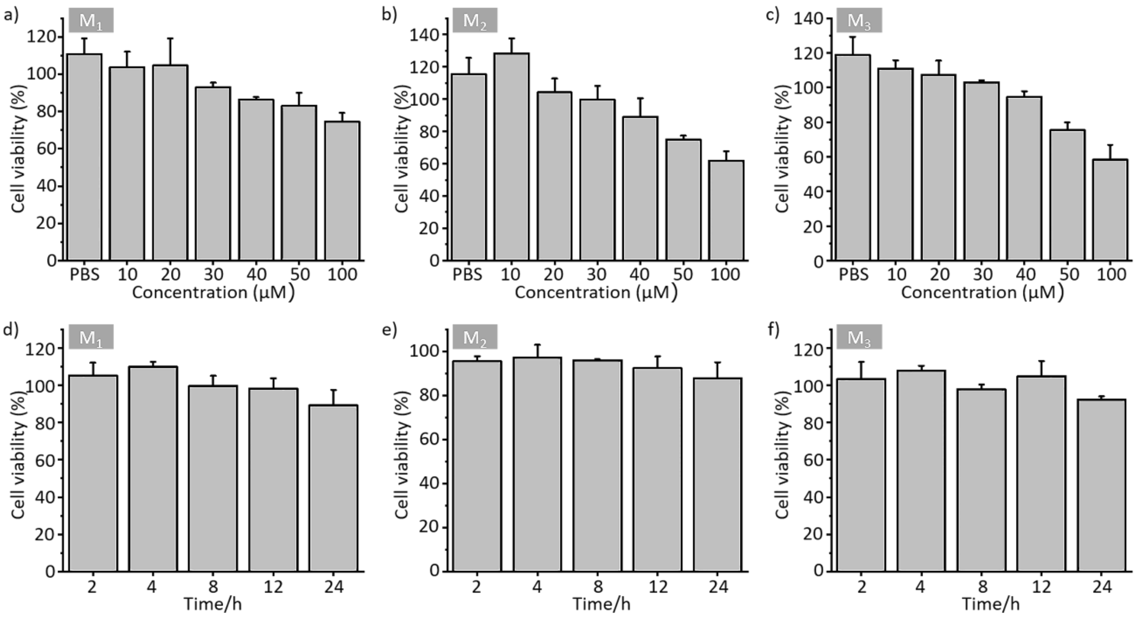

Figure S10. The cell viability of HUVECs treated by $\mathbf{M_1}$ (a), $\mathbf{M_2}$ (b) and $\mathbf{M_3}$ (c) with different concentration. The cell viability of HUVECs incubated with $\mathbf{M_1}$ (d), $\mathbf{M_2}$ (e) and $\mathbf{M_3}$ (f) for different time at the concentration of 20 μM. The $\mathbf{M_1}$-$\mathbf{M_3}$ showed good biological safety.

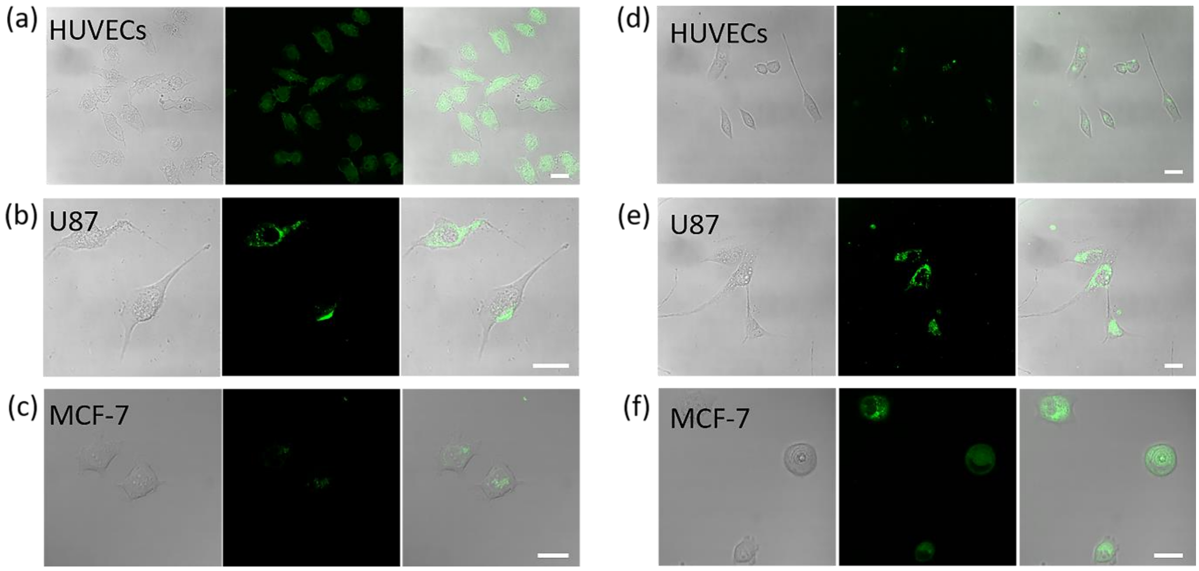

Figure S11. The CLSM images of HUVECs (a), U87 cells (b) and MCF-7 cells (c) treated with $\mathbf{M_2}$ for 24 h; The CLSM images of HUVECs (d), U87 cells (e) and MCF-7 cells (f) treated with $\mathbf{M_3}$ for 24 h. Scale bar: 10 μm.

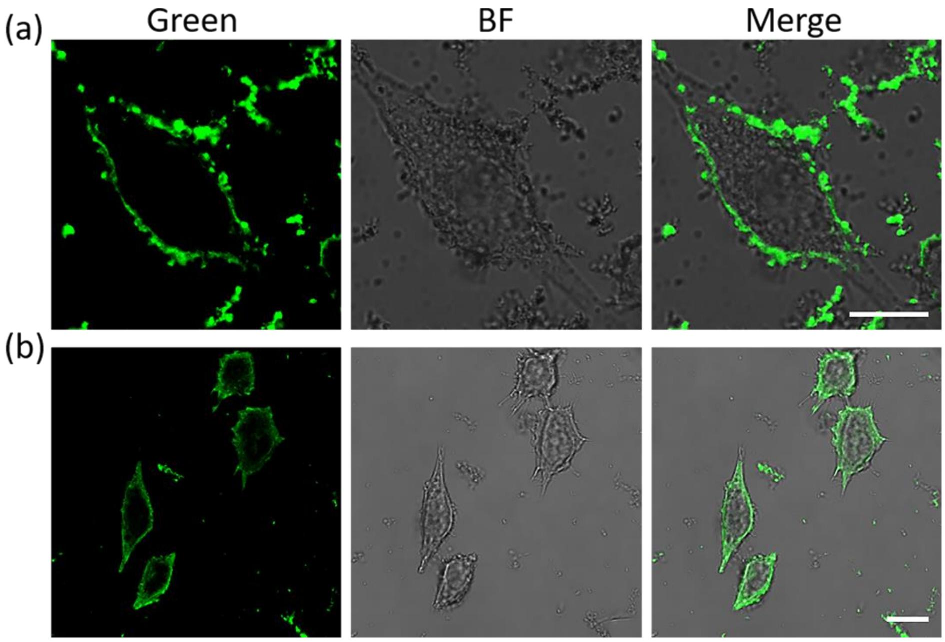

Figure S12. The CLSM images of HUVECs treated with $\mathbf{M_1}$ for 4 h (a) and 8 h (b). Scale bar: 10 μm.

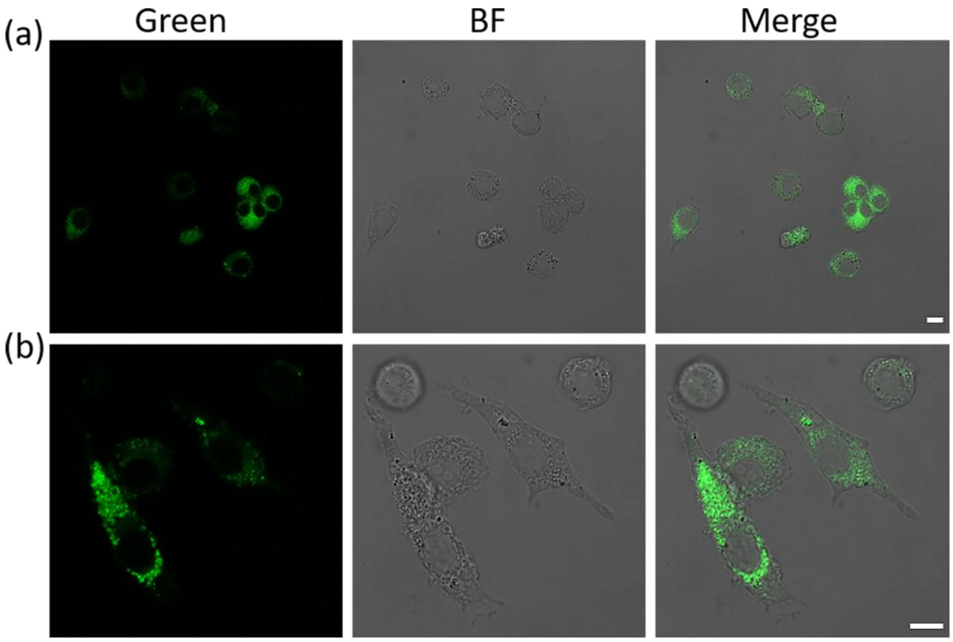

Figure S13. The CLSM images of HUVECs treated with $\mathbf{M_2}$ for 4 h (a) and 8 h (b). Scale bar: 10 μm.

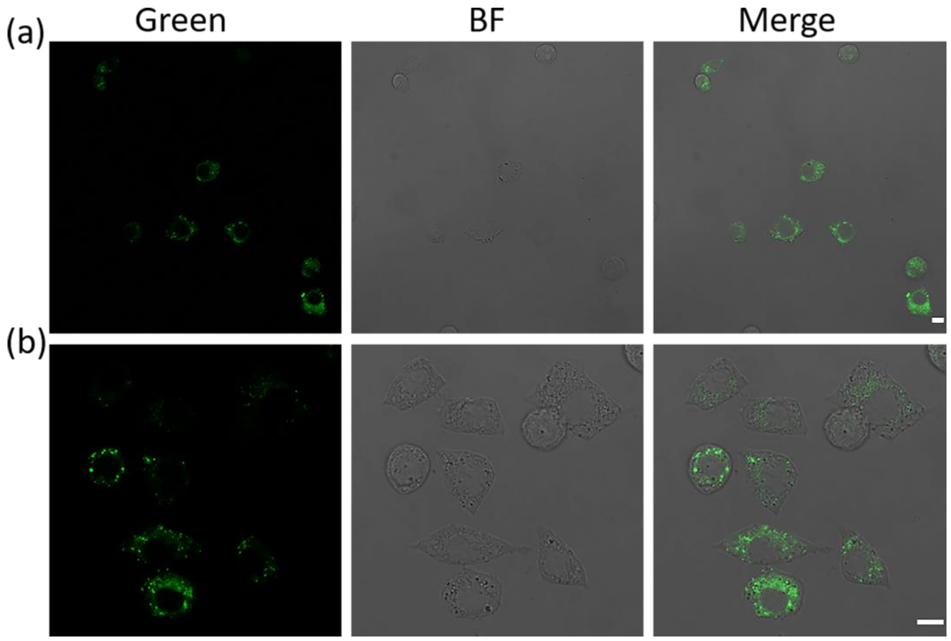

Figure S14. The CLSM images of HUVECs treated with $\mathbf{M_3}$ for 4 h (a) and 8 h (b). Scale bar: 10 μm.

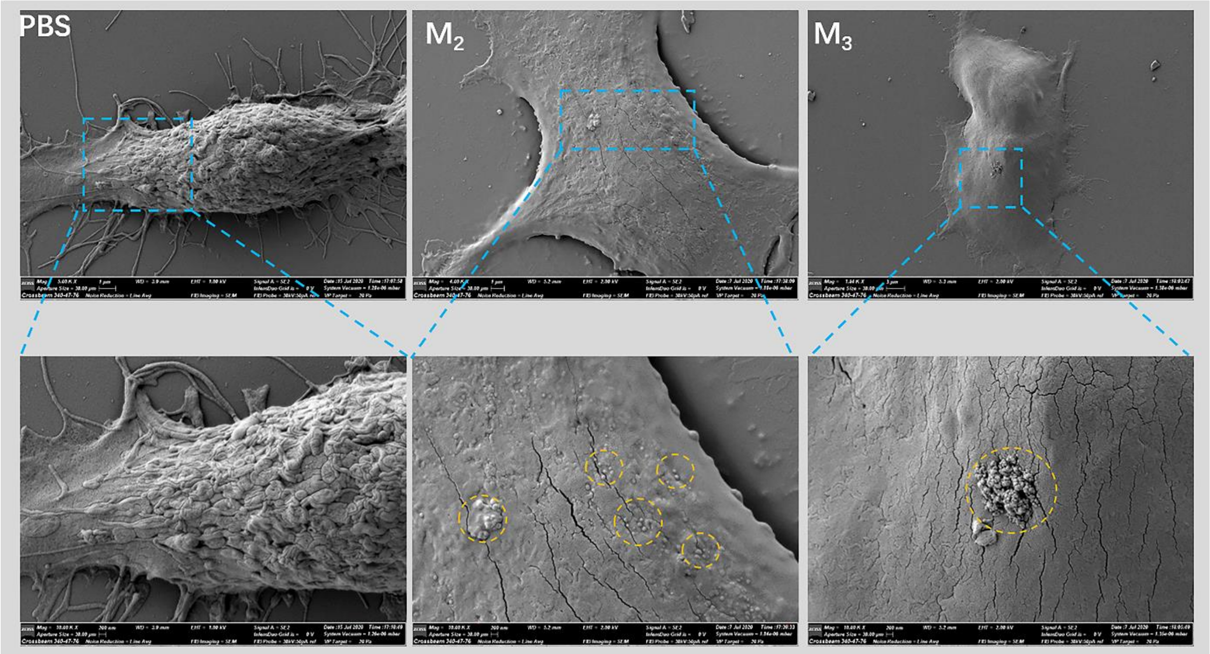

Figure S15. SEM images showing PBS, $\mathbf{M_2}$ and $\mathbf{M_3}$ in HUVECs.

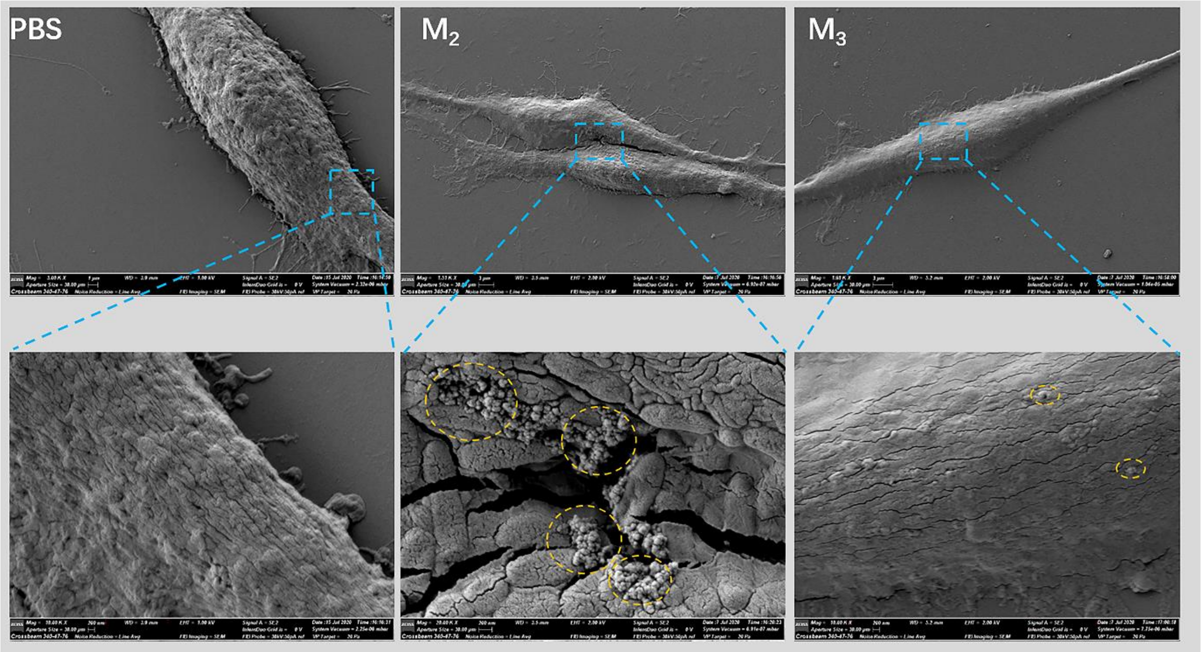

Figure S16. SEM images showing PBS, $\mathbf{M_2}$ and $\mathbf{M_3}$ in U87 cells.

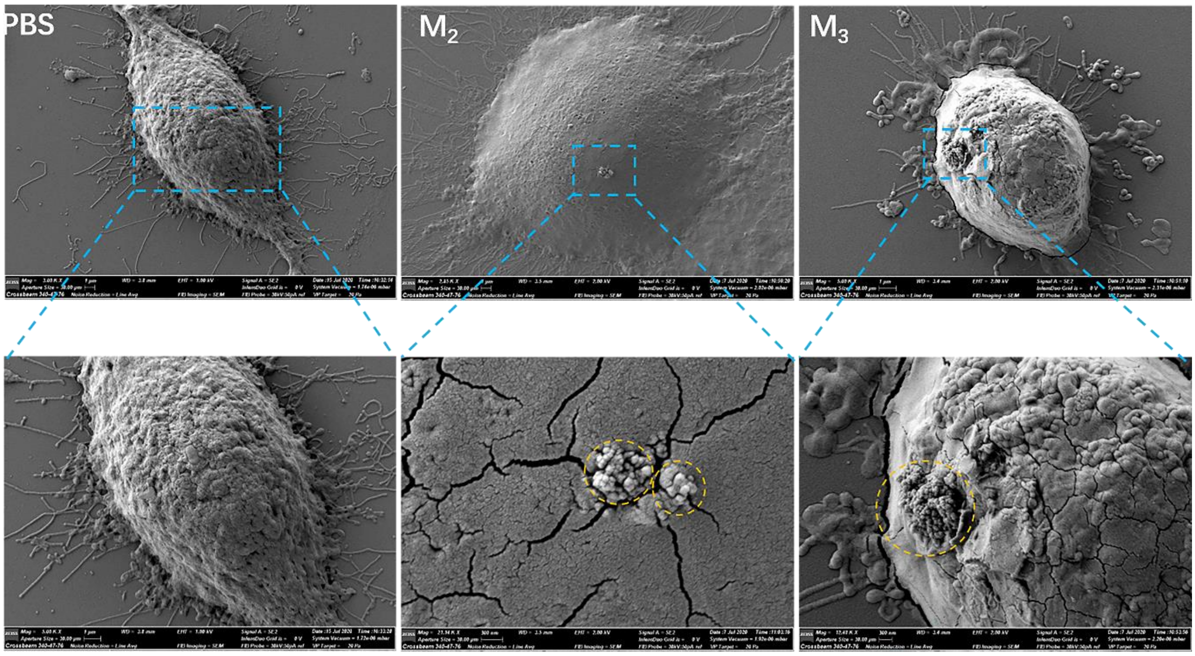

Figure S17. SEM images showing PBS, $\mathbf{M_2}$ and $\mathbf{M_3}$ in MCF-7 cells.

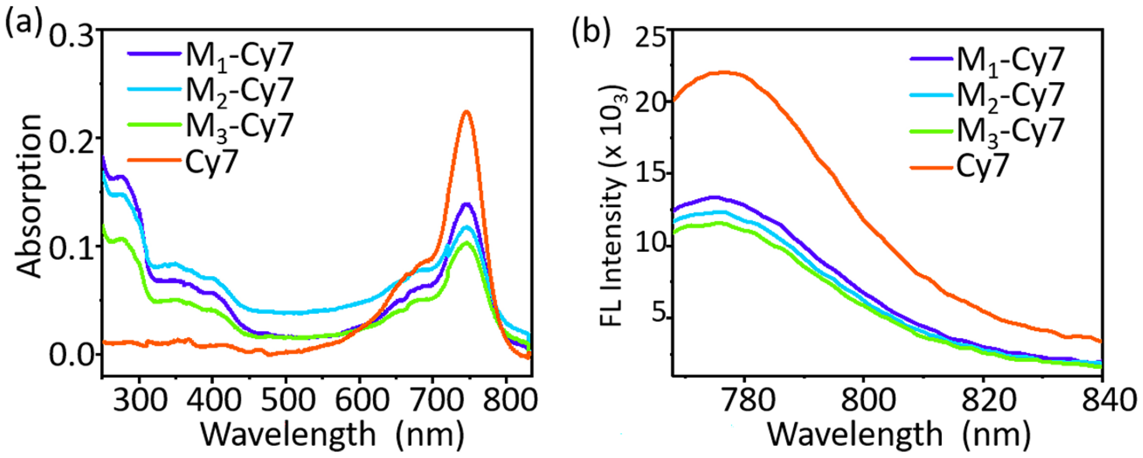

Figure S18. $\mathbf{M_1}$, $\mathbf{M_2}$ and $\mathbf{M_3}$ were labeled with Cy7-NHS to obtain $\mathbf{M_1}$-, $\mathbf{M_2}$- and $\mathbf{M_3}$-Cy7. (a) The UV spectrum analysis of free Cy7, $\mathbf{M_1}$-, $\mathbf{M_2}$- and $\mathbf{M_3}$-Cy7. (b) The fluorescence assay of free Cy7, $\mathbf{M_1}$-, $\mathbf{M_2}$- and $\mathbf{M_3}$-Cy7 (λ_{ex} = 749 nm, λ_{em} = 765-850 nm).

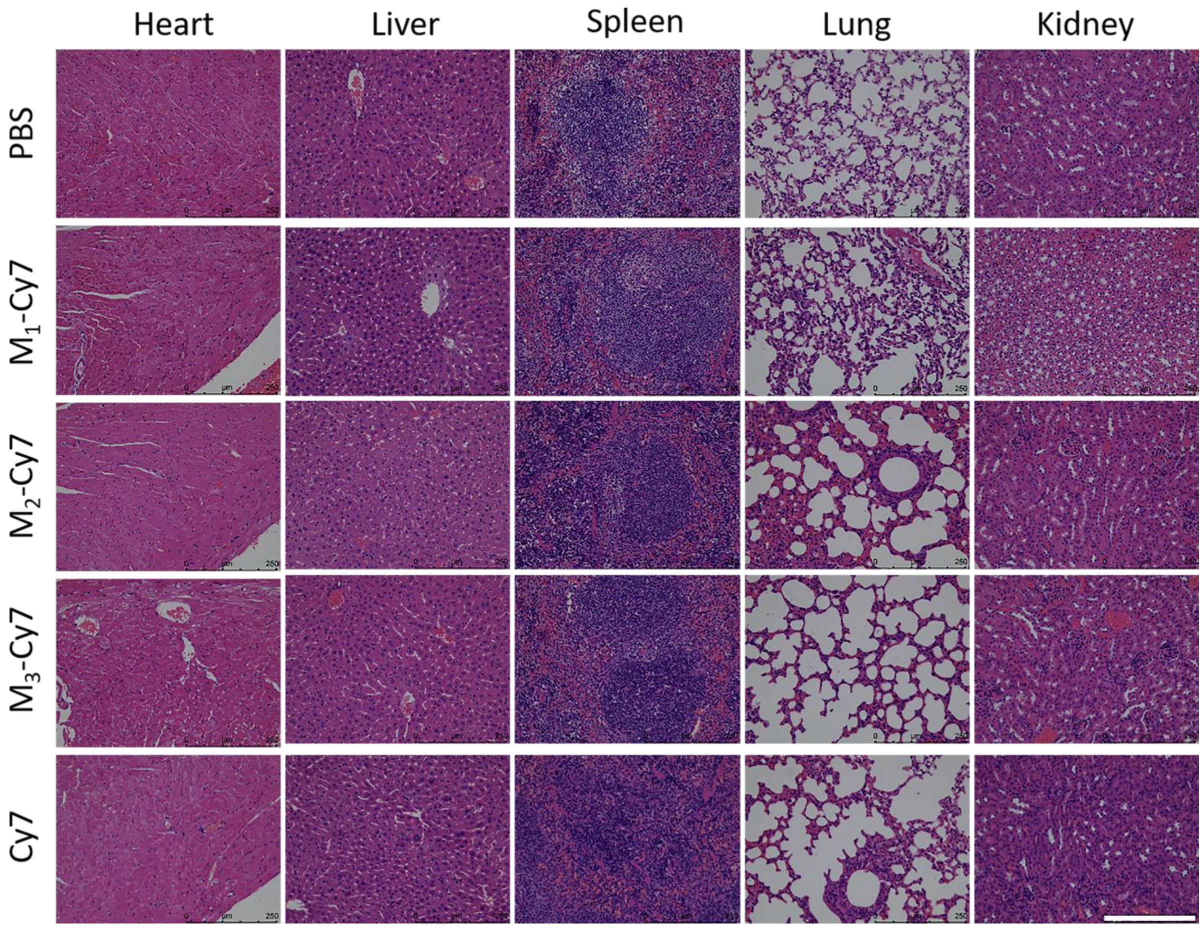

Figure S19. Histology evaluation of major organs (heart, liver, spleen, lung and kidney) after treatment with PBS, **M$_1$**-Cy7, **M$_2$**-Cy7 and **M$_3$**-Cy7 and free Cy7. Scale bar: 250 μm.
